# miR-296-5p Inhibits the Secretion of Pulmonary Surfactants in Pulmonary Epithelial Cells via the Downregulation of Wnt7b/*β*-Catenin Signaling

**DOI:** 10.1155/2021/4051504

**Published:** 2021-01-05

**Authors:** Ying-Hui Zhang, Ai-Ling Chen, Ren-Qiang Yu, Bei-Bei Jia, Dan-Ni Ye, Min Wang, Ying-Zi Mei, Guang-Dong Fang, Shan-Yu Jiang, Qin Zhou, Bing Zhang

**Affiliations:** ^1^Department of Neonatology, The Affiliated Wuxi Maternity and Child Health Care Hospital of Nanjing Medical University, Wuxi, China; ^2^Translational Medicine Laboratory, Research Institute for Reproductive Health and Genetic Diseases, The Affiliated Wuxi Maternity and Child Health Care Hospital of Nanjing Medical University, Wuxi, China; ^3^Department of Gynaecology and Obstetrics, The Affiliated Wuxi Maternity and Child Health Care Hospital of Nanjing Medical University, Wuxi, China

## Abstract

Neonatal respiratory distress syndrome (NRDS) is a common disease that occurs in premature infants. However, the mechanisms underlying the disease remain unclear. microRNAs (miRNAs) have been indicated to play a crucial role in the development of NRDS. In this study, we aimed to explore the regulatory mechanisms of miR-296-5p in NRDS. The expression levels of miR-296-5p in preterm infants with NRDS were determined using quantitative reverse-transcription polymerase chain reaction (RT-qPCR). A549 cells were transfected with lentiviral vectors encoding miR-296-5p, and the transfection efficiency was determined using RT-qPCR. Flow cytometry and CCK8 assay were performed to measure apoptosis and proliferation of A549 cells, respectively. The protein levels of pulmonary surfactant SP-A (SFTPA1), SP-B, Wnt7b, and *β*-catenin were measured using western blotting. We demonstrated an upregulation of miR-296-5p in NRDS. The miR-296-5p was successfully overexpressed in A549 cells via lentivirus transfection, and the upregulation of miR-296-5p inhibited cell proliferation and secretion of SP-A and SP-B and also induced downregulation of the Wnt7b/*β*-catenin *in vitro*. Therefore, miR-296-5p inhibits cell proliferation and secretion of pulmonary surfactants in A549 cells via downregulation of Wnt7b/*β*-catenin signaling.

## 1. Introduction

Neonatal respiratory distress syndrome (NRDS) is a serious disease that occurs in approximately 45% of mildly to moderately premature infants, which is associated with high morbidity and mortality. The major cause of this disease is lack of alveolar surfactant leading to a collapse in alveolar ventilation, which in turn gives rise to progressive dyspnea and even respiratory failure within 4–12 h after birth [1]. Steroid prophylactic treatment and exogenous pulmonary surfactant (PS) replacement therapy are used in treating NRDS; however, the clinical efficacy of these strategies is still not satisfactory. Clinical medicine, epidemiology, and biochemistry have demonstrated that NRDS is associated with various internal and external factors such as lung dysplasia, severe infection, hypoxia, and direct damage to type II alveolar epithelial cells (AECII). Clinically, the amniotic fluid foam test, lecithin/sphingomyelin (L/S), phosphatidylglycerol determination, lamellar body count (LB), alveolar surfactant/albumin detection, and lung maturity predict the occurrence of NRDS. However, these invasive techniques are complicated, and the false positive and negative error rates associated with these techniques are high, thereby affecting the clinical efficacy. Therefore, the technique to identify the risk for NRDS must be improved to predict the onset of NRDS in infants.

microRNAs (miRNAs), small noncoding RNAs of ~22 nucleotides long, are known to be involved in the regulation of various cellular processes [2], such as cell differentiation and development, and may also be associated with numerous diseases [3], acting as cancer oncogenes [4]. Many studies have demonstrated that >100 miRNAs change in expression levels during lung development [5]. However, the mechanism for miRNA regulation of lung development remains unclear. Recent research has shown that miRNAs are differentially expressed in embryonic and adult lung tissues [6–8]. A study demonstrated that the inactivation of Dicer, a key component of miRNA processing, inhibits alveolar epithelial cell differentiation [9]. miRNA-17-92 overexpression has been demonstrated to promote alveolar epithelial cell proliferation and inhibit its differentiation [10]. Another study showed that miRNA-155 increased lung remodeling in knockout mice [11]. miRNA-127 overexpression leads to decreased fetal apical bud number and increased terminal and internal bud size, affecting fetal lung development [7]. These results suggest that miRNAs are closely related to lung development and may be involved in the development of NRDS. However, the role of miRNAs in NRDS has not been extensively studied. In our previous study [12], we found that the expression of miR-296 was different in fetal mice at 16 days, 19days, and 23days. However, whether miR-296 is involved in lung development remains unclear. The current research on miR-296 mainly focuses on tumors, including the proliferation of tumor cells [13–18], differentiation [14], angiogenesis [19–21], invasion [14, 22, 23], and drug resistance [24–26]. There are no reports on the role of miR-296 in NRDS. However, the literature demonstrates that miR-296 is also involved in the biological processes of embryonic stem cells. It has been found that miR-296 can inhibit the nuclear stem cell gene, Nanog [27], and is significantly upregulated in epithelial cells derived from human embryonic stem cells [28]. The study found that AECII are the main stem cells present in the lungs [29]. In the mechanism of normal cell renewal and injury repair, AECII can replenish and proliferate via mitosis and can also be transdifferentiated into type I alveolar epithelial cells. However, there are no reports on their function and mechanism in the development of lung tissue. Therefore, we hypothesized that miR-296 may participate in regulating the development of AECII and contribute to the NRDS process.

In our previous study [12], three major target gene software: TargetScan6.2, PicTar, and miRanda, were used for performing functional and pathway enrichment analyses of miR-296. Downstream target genes and signaling pathways suggest that the Wnt signaling pathway is a major signaling pathway enriched with miR-296 target genes. Therefore, miR-296 may be involved in the regulation of the Wnt signaling pathway. The Wnt signaling pathway is widely distributed in invertebrates and vertebrates and is a highly evolutionarily conserved signaling pathway. Wnt signaling plays a crucial role in physiological processes, such as the early development of animal embryos [30], organ formation [31], and tissue regeneration [32]. Binding of the Wnt molecule to a transmembrane receptor triggers the accumulation of *β*-catenin in the cytoplasm and its subsequent translocation into the nucleus via interaction of the receptor with Dishevelled (DVL) and a series of cytoplasmic proteins [33]. Further, nuclear transcription factors activate the transcription of downstream target genes, triggering biological effects [34]. Further studies have shown that the Wnt signaling pathway is not only crucial in embryonic development but is closely associated with the self-renewal and differentiation of various tissue stem cells. The Wnt signaling pathway is also implicated in the occurrence and development of various human diseases [35, 36]. During lung development, blocking the Wnt signaling pathway via gene knockout of *β*-catenin blocks the formation of distal branches of the lungs and the differentiation of terminal airway epithelial cells, resulting in NRDS [37]. These results indicate that the Wnt signaling pathway is involved in the pathogenesis of NRDS. Therefore, we hypothesized that miRNA-296 may play an important role in NRDS by interacting with Wnt and modulating lung development, which in turn affects the formation of PS. The synthesis and secretion of PS play a crucial role in NRDS. This study may provide novel theoretical clues for the clarification and prevention of NRDS pathogenesis.

## 2. Materials and Methods

### 2.1. Patients and Sample Collection

The study protocol was approved by the Human Research Ethics Committee of the Affiliated Wuxi Maternity and Child Health Care Hospital of Nanjing Medical University. Written informed consent was obtained from all pregnant women involved in the study. Venous cord blood (2 mL) was collected during the birth of 23 preterm infants (13 without NRDS, 10 with NRDS). Serum samples were obtained from the clotted cord blood specimens and stored in a -80°C ultralow freezer until further use.

### 2.2. Cell Culture

Cells of the A549 human lung adenocarcinoma cell line were purchased from Shanghai GeneChem Co., Ltd. (Shanghai, China) and cultured in DMEM containing 10% fetal bovine serum and 1% penicillin-streptomycin solution (Gibco, Thermo Fisher Scientific, Waltham, MA, USA). The cells were maintained in an incubator in an atmosphere containing 5% CO_2_ at 37°C. All the experiments in this study were repeated at least three times.

### 2.3. Construction of Plasmids

A lentiviral GV369 vector was purchased from Shanghai GeneChem Co., Ltd. The vector sequence was Ubi-MCS-SV40-EGFP-IRES-puromycin. The primer sequences of miR-296-5p were as follows: forward, 5′-GAGGATCCCCGGGTACCGGGGACAGGGCTGGGAGGATTGAG-3′; and reverse, 5′-CACACATTCCACAGGCTAGGCCGCCCCAGGGGACTCAGCAG-3′. The primers contained complementary base pairs, restriction sites, and part of the 5′-end sequence of the target gene for PCR. Subsequently, according to the manufacturer's protocol, the designed vector was digested by AgeI and NheI restriction enzymes at 37°C for 30 min simultaneously and ligated by DNA ligase at 16°C for 2 h. The recombinant plasmid was validated by double restriction enzyme digestion, separated by 1% PAGE, and sequenced by Sanger sequencing by Sangon Biotech Co., Ltd. The correct recombinant plasmid was termed named pGV369-296-5p.

### 2.4. Lentivirus Packaging

To generate lentivirus containing the recombinant plasmids, HEK 293 T cells were cultured in a cell culture plate until they reached 70-80% confluence. The media were then replaced with serum-free medium and incubated for 2 h. Further, the cells were transfected with 20 *μ*g of the recombinant plasmid, pGV369-296-5p, along with helper plasmids, pHelper 1.0 vector plasmid (15 *μ*g) and pHelper 2.0 vector plasmid (10 *μ*g). The cells were washed with phosphate-buffered saline (PBS) 6 h posttransfection, and the media were replaced with DMEM containing 10% FBS. The media of HEK 293 T cells were collected 48 h after transfection, and the virus (L-GV369-296-5p) was harvested. The supernatant was filtered through a 0.45 *μ*m filter and centrifuged at 25,000 rpm for 2 h. The supernatant was discarded, and the pellet containing the virus was resuspended with a virus preservation solution. The solution was then centrifuged at 10,000 rpm for 5 min, and the supernatant was stored in a -80°C ultralow freezer.

### 2.5. Lentivirus Infection

The A549 cells were seeded on a 24-well plate at a density of 5–8 × 10^4^ cells/well and infected with 1 × 10^8^ TU/mL, 1 × 10^7^ TU/mL, and 1 × 10^6^ TU/mL for 48 h. A control cell line (NC-A549) without lentivirus infection was also maintained. The stable transfected cells were then screened using puromycin. Trypsin was used to dissociate cells into a single-cell suspension. The cells were then seeded onto a 96-well plate to obtain a cellular monolayer in order to establish cell lines.

### 2.6. Cell Viability Assay

A549 cells were seeded onto 96-well plates at a density of 7,000 cells/well and maintained for 48 h. Following which, the CCK-8 solution (Dojindo, Japan) was carefully added to the cells (100 *μ*L per well). The plates were then incubated in the dark for 2 h at 37°C. The absorbance was periodically detected at 450 nm using a microplate reader.

### 2.7. Cell Apoptosis Assay

A549 cell apoptosis assay was performed via flow cytometry using an Annexin V/PI Apoptosis Kit (BD, Franklin Lakes, NJ, USA). A549 cells were collected following the transfection of miR-296-5p lentiviral vector or empty lentiviral vector for 48 h; then, these cells were then labeled using Annexin V and PI according to the manufacturer's instruction. Flow cytometry assay was performed to detect the percentage of early apoptotic cells.

### 2.8. RT-qPCR Analysis for RNA Expression

Total RNA was extracted using TRIzol (Invitrogen, Carlsbad, CA, USA) according to the manufacturer's instruction. RT-qPCR was performed using Fast Start Universal SYBR Green Master (Roche). U6 was used as an endogenous control. Fold changes in the mRNA expression levels normalized to GAPDH were calculated using the 2^-*ΔΔ*ct^ method. The following primers were used: U6 forward, 5′-CTCGCTTCGGCAGCACA-3′; U6 reverse, 5′-AACGCTTCACGAATTTGCGT-3; GAPDH forward, 5′-CCTCAAGATCATCAGCAATGCCTC-3′; GAPDH reverse, 5′-GTGGTCATGAGTCCTTCCACGATA-3′; miR-296-5p forward, 5′-TGCCTAATTCAGAGGGTTGG-3′; and miR-296-5p reverse, 5′-CTCCACTCCTGGCACACAG-3′.

### 2.9. Protein Extraction and Western Blotting

The A549 cells were cultured for 48 h. The cells were then washed twice with PBS. Radioimmunoprecipitation assay buffer (RIPA; Beyotime, Shanghai, China) containing 1 mM phenylmethanesulfonylfluoride (PMSF; Beyotime, Shanghai, China) was added, and the cells were detached using a cell scraper. The solution was then centrifuged for 20 min at 12,000 rpm at 4°C and further lysed using ultrasound. Protein concentrations were quantified using the BCA protein assay kit (Beyotime, Shanghai, China). Cell lysates were boiled for 10 min at 95°C and stored at -80°C. Total proteins (20 *μ*g) were electrophoresed on SDS-PAGE gels using the Miniprotein III system (Bio-Rad, California, USA) and were then transferred to PVDF membranes (Millipore, USA) for 2 h, followed by overnight incubation with primary antibody against WNT7B, Catenin-beta, SFTPA1 (SP-A), SP-B, or GAPDH (1 : 1000; Affinity Biosciences, Cincinnati, OH, USA) at 4°C. The following day, PVDF membranes were washed three times using PBST solution and were incubated at room temperature for 1 h in peroxidase-conjugated goat antirabbit IgG secondary antibodies (1 : 5,000; Bioworld Technology, Co. Ltd., USA). Further, the membrane was washed three times and processed for chemiluminescence using the Immobilon Western Chemiluminescent HRP Substrate Kit (Millipore, USA).

### 2.10. Statistical Analysis

Numerical data were reported as mean ± SD. The GraphPad Prism version 5 software program was used for statistical analyses. Continuous variables were analyzed using Student's *t*-test or Mann–Whitney *U* test to compare differences among groups. *p* < 0.05 was considered significant.

## 3. Results

### 3.1. miR-296-5p Was Overexpressed in the Serum of Premature Infants with NRDS

**Figure 1:**
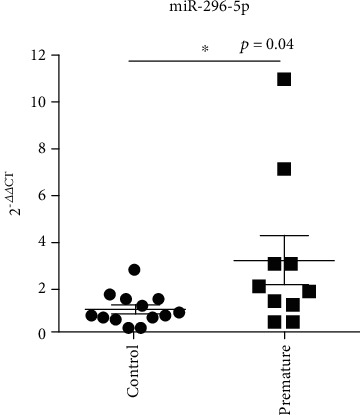
miR-296-5p was highly expressed in the sera of premature infants with NRDS. The sera from 13 premature infants without NRDS and 10 premature infants with NRDS were collected, and then, RT-qPCR assay was used to detect miR-296-5p expression. ^∗^*p* < 0.05.

To determine the expression levels of miR-296-5p in preterm infants with NRDS, we collected the serum samples from 13 preterm infants without NRDS and 10 preterm infants with NRDS. As shown in Figure 1, the values of miR-296-5p were 1.04 ± 0.69 and 3.16 ± 3.35, respectively, and the difference between the two groups was statistically significant (*p* = 0.04), indicating the overexpression of mir-296-5p in NRDS preterm infants as compared to non-NRDS preterm infants.

### 3.2. Establish Overexpression of miR-296-5p in A549 Cell Lines

**Figure 2:**
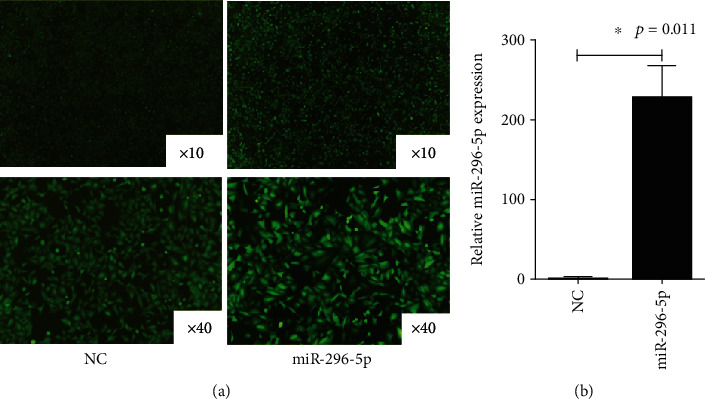
Lentiviral vector-mediated transfection was used to establish stable overexpression of miR-296-5p in A549 cell lines. (a). The transfection efficiency of lentivirus mir-296-5p in A549 cells was detected using fluorescence. (b). The RT-qPCR assay was used to detect miR-296-5p expression after transfection. ^∗^*p* < 0.05.

To elucidate the role of miR-296-5p in NRDS, we constructed lentiviral miR-296-5p overexpression cell lines (hereafter “miR-296-5p group”). Following the transfection of the miR-296-5p lentiviral vector into A549 cells, fluorescence microscopy demonstrated that the transfection rate was greater than 85%, as shown in Figure 2(a). The expression levels of miR-296-5p in NC-A549 and A549 cells were detected using RT-qPCR. The expression of miR-296-5p in the miR-296-5p group was significantly higher than that in the NC-A549 group (*p* = 0.011) (Figure 2(b)).

### 3.3. miR-296-5p Inhibited the Proliferation of A549 Cells with Unchanged Apoptosis

**Figure 3:**
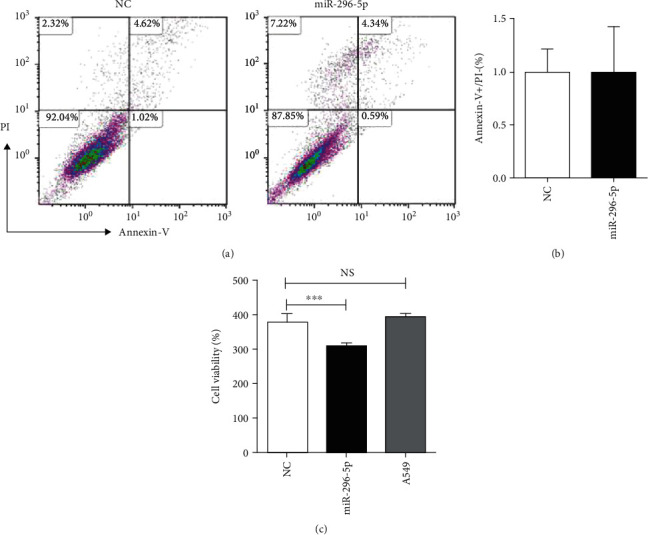
miR-296-5p inhibited proliferation of A549 cells with unchanged apoptosis. (a), (b). The apoptosis of A549 cells was analyzed using the apoptosis assay. (c). The cell viability of A549 cells was detected using the CCK-8 assay. ^∗∗∗^*p* < 0.001.

To study the effect of miR-296-5p on the A549 cells, we cultured the overexpressed miR-296-5p, NC-A549, and A549 cells for 24, 48, and 72 h. Further, apoptosis and proliferation of A549 cells were examined using flow cytometry and CCK8 assay, respectively. The results of the Annexin-V/PI apoptosis assay showed that the percentage of apoptotic cells (Annexin-V+PI-) was 0.9% in the miR-296-5p group and 1% in the NC group (*p* > 0.05) (Figures 3(a) and 3(b)). The data of the CCK8 assay showed that cell viability was significantly lower in the miR-296-5p group compared to the NC group (*p* < 0.001) (Figure 3(c)). These results suggest that miR-296-5p may inhibit the proliferation of A549 cells. However, miR-296-5p inhibition did not induce apoptosis *in vitro*.

### 3.4. miR-296-5p May Inhibit the Expression of SP-A and SP-B through the WNT Signaling Pathway

**Figure 4:**
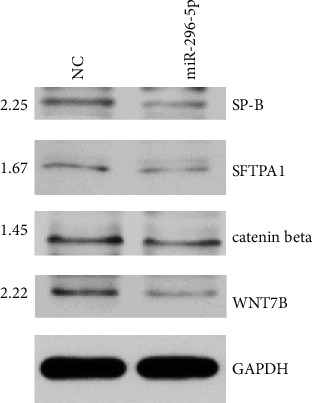
miR-296-5p inhibited the expression of SP-A (SFTPA1) and SP-B through the Wnt signaling pathway. After transfection of mir-296-5p overexpression and empty vectors in the A549 cell line, the expression of WNT7B, *β*-catenin, SP-A, and SP-B was assessed using western blotting.

PS plays a crucial role in the maintenance of normal lung function in neonates. Lack of PS has been implicated in NRDS. Surfactant protein A (SP-A, SFTPA1) and surfactant protein B (SP-B) are the most important components of PS. To explore the potential mechanism of miR-296-5p in A549 cells, we determined the ratio of SP-A to SP-B among the NC and miR-296-5P groups via western blotting and found them to be 1.67 and 2.25, respectively (Figure 4). These results indicate that miR-296-5p inhibits the production of SP-A and SP-B in A549 cells. Wnt7b is one of the target genes of miR-296-5p. Therefore, in this study, we investigated the role of the Wnt7b/*β*-catenin pathway in NRDS using western blot. The ratios of Wnt7b and *β*-catenin among the NC and miR-296-5p groups were 2.22 and 1.45, respectively (Figure 4). These results suggest that miR-296-5p may inhibit SP-A and SP-B through the Wnt7b pathway in A549 cells.

## 4. Discussion

PS is primarily composed of phospholipids that could reduce alveolar surface tension and maintain the effective exchange of blood gases. Surfactant proteins SP-A, SP-B, SP-C, and SP-D are known to be crucial components of alveolar surfactant. Each of them makes contributions to lung homeostasis through their primary protein structures and activities [38]. Mutations in the genes encoding SP-B (*SFTPB*) and SP-C (*SFTPC*) have been identified as pathogenic factors in full-term infants with refractory respiratory failure after birth [39, 40]. NRDS is pathologically characterized by progressive dyspnea and respiratory failure due to inadequate secretion of surfactant lipids and proteins by immature AECII. Expanding the knowledge of surfactant proteins and lipids can elucidate improved applications to prevent and treat NRDS.

Recent studies have shown that miRNAs are involved in the developmental process of lung maturation. Bhaskaran et al. identified significant changes in expression of 21 miRNAs during lung development with a miRNA microarray and demonstrated that miR-127 had the greatest expression during the late stage of fetal lung development [7]. The expression of miR-431 was demonstrated to decline in preterm infants without RDS compared with those with RDS [41]. *In vitro* cell experiments show that miR-431 restricts the expression of SP proteins through suppression of the BMP4/activin/transforming growth factor-*β* signaling pathway by targeting SMAD4 in A549 cells [42]. In the present study, the expression of miR-296-5p in preterm infants with NRDS was greater than in premature infants without NRDS. The sample size was small however; thus, we need to expand the sample size in future experiments. Würdinger et al. [21] demonstrated that miR-296-5p promotes the expression of growth factor receptors in vascular epithelial cells and affects tyrosine kinase substrates via regulation of hepatocyte growth factor. This results in a reduced number of blood vessels and induction of degradation of receptors for endothelial growth factor and platelet-derived growth factor. We found the ratio of SP-A to SP-B in the NC and miR-296-5P groups to be 1.67 and 2.25, respectively. This suggests that miR-296-5p may inhibit the expression of SP proteins in A549 cells.

Wnt/*β*-catenin signaling plays an important role in the differentiation and development of fetal lungs [43, 44]. Clevers et al. [45] demonstrated that Wnt signaling in the mouse- and human-induced pluripotent stem cells is an effective regulator of proximal and distal epithelial patterns. Wnt7b is mainly located in proximal and distal bronchial epithelial cells [46], and Wnt7b downstream *β*-catenin is restricted in alveolar and airway epithelial cells [47]. Murine model studies have shown that Wnt/*β*-catenin signaling is imperative for the proliferation of airway submucosal gland progenitor cells, and the deletion and/or worsening of Wnt/*β*-catenin signaling in the early development of lung epithelium cells leads to severe disruption of mesenchymal and epithelial compartments. This subsequently leads to the attenuation of secondary and tertiary branching [47] and hyperplasia and hypertrophy of submucosal glands [48]. However, in the present study, the ratios of Wnt7b and *β*-catenin in the NC and miR-296-5p groups were 2.22 and 1.45, respectively. This suggests that miR-296-5p may inhibit Wnt/*β*-catenin signaling in A549 cells.

There are some limitations to our study. AECII have certain stem cell characteristics including the ability to automatically transdifferentiate during *in vitro* culture. The intervention conditions of AECII are complex, which does not provide sufficient time to carry out the study accordingly. Therefore, we introduced a cell line, A549, as a replacement for AECII. The A549 cell line is a classic human AECII, containing lamellar bodies to produce surfactant and has a phospholipid content similar to that of AECII in situ [49]. We only detected proliferation and pulmonary surfactant secretion by A549 cells after transfection of the cells with miR-296-5p. According to our studies to date, we suggest that miR-296-5p could be involved in the occurrence and development of NRDS via Wnt/*β*-catenin signaling. However, we did not assess the status of other miR-296-5p target genes as well. We also did not further verify the biological function of miR-296-5p *in vivo*. These limited findings require further studies to clarify the underlying molecular mechanism.

## 5. Conclusion

In summary, the present study revealed that miR-296-5p levels were elevated in NRDS, which suppressed the proliferation and secretion of pulmonary surfactants in A549 cells by downregulating Wnt7b/*β*-catenin signaling. Hence, the results of the present study hint at a novel molecular mechanism in which miR-296-5p is overexpressed in NRDS thereby deactivating Wnt7b/*β*-catenin signaling.

## Data Availability

The datasets used and/or analyzed during the current study are available from the corresponding author on reasonable request.
